# Deep Learning Based Muti-Objective Reactive Power Optimization of Distribution Network with PV and EVs

**DOI:** 10.3390/s22124321

**Published:** 2022-06-07

**Authors:** Renbo Wu, Shuqin Liu

**Affiliations:** School of Electrical Engineering, Shandong University, Jinan 250061, China; lshuqin@sdu.edu.cn

**Keywords:** photovoltaic, electric vehicles, reactive power optimization, deep learning, Pareto front

## Abstract

With the high penetration of photovoltaic (PV) and electric vehicle (EV) charging and replacement power stations connected to the distribution network, problems such as the increase of line loss and voltage deviation of the distribution network are becoming increasingly prominent. The application of traditional reactive power compensation devices and the change of transformer taps has struggled to meet the needs of reactive power optimization of the distribution network. It is urgent to present new reactive power regulation methods which have a vital impact on the safe operation and cost control of the power grid. Hence, the idea that applying the reactive power regulation potential of PV and EV is proposed to reduce the pressure of reactive power optimization in the distribution network. This paper establishes the reactive power regulation models of PV and EV, and their own dynamic evaluation methods of reactive power adjustable capacity are put forward. The model proposed above is optimized via five different algorithms and approximated through the deep learning when the optimization objective is only set as line loss and voltage deviation. Simulation results show that the prediction of deep learning has an incredible ability to fit the Pareto front that the intelligent algorithms obtain in practical application.

## 1. Introduction

Renewable energy sources (RES) such as the photovoltaic (PV) system have played an important part in reducing environmental pollution in recent years due to their ability to reduce greenhouse effects [1]. As a kind of mature and widely used power generation method, PV power generation perfectly conforms to the strategy of sustainable development and the concept of safe power generation. With the development of distributed generations (DGs), PV can be operated at a smaller scale called distributed energy resources (DER). This form of PV aims to be closer to the load that needs to consume power, which uses the idea of decentralized investment to reduce the loss in the transmission [2]. However, PV has the characteristics of intermittence and instability. Solar irradiation, cloud cover, photovoltaic panel orientation or dust diffusion and other factors may interfere with the normalized PV to a great extent [3]. Besides, high penetration of PV may lead to the issues of voltage rise, reverse power flow, and increased energy loss [4].

Nevertheless, with the rapid development of electric vehicles (EVs) in recent years, it is becoming increasingly possible to alleviate this negative phenomenon. Some studies show that EVs can not only carry out routine charging operation, but also profitably provide power to the power grid after the EVs with new technology called vehicle-to-grid (V2G) are connected to the power supply [5]. Therefore, a new idea for implementing energy storages to the grid which has a strong impact on the traditional distribution network, and EVs are gradually being accepted by people [6]. V2G greatly improves the flexibility and availability of EVs. In general, PV is greatly affected by time changes. The energy storage of EVs can solve the problems of power overflow and voltage rise caused by PV at noon, and realize the operation of power transmission to the power grid at the peak of power consumption at night [7]. In addition, in response to the worldwide appeal to reduce carbon emissions, the use of EVs is expected to become more widespread. This trend caters to the need to solve the problems caused by new energy generation in the distribution network.

Some research has shown that when distributed PV and EVs are connected to the distribution network, reactive power optimization of distribution system will be a complex discrete, nonconvex and nonlinear problem [8]. In the daily operation of the distribution network, line loss of transmission and voltage deviation are usually the issues that need attention. It is necessary to optimize the variables with line loss minimization and voltage deviation minimization as two different objectives. The classical methods commonly used by researchers to solve such multi-objective problems (MOPs) are the interior point method and the Newton method. The idea of these methods is to transform the MOPs into some single objective optimization problems with assignable weight. The problem highlighted by these methods is that the setting of weight is highly subjective and may not reach a balance point. Using the multi-objective evolution algorithm (MOEA) to solve MOPs is also a good choice for current optimization problems, but these methods usually spend a lot of time on optimization in the face of complex problems.

The characteristics of PV and EVs show the ability to provide reactive power support for a distribution network. As a premise, the future development trend of different types of EVs was well estimated in reference [9]. With the combination of a residential roof PV system and EVs, the authors in [10] demonstrated the potential and technical benefits of such system in terms of the reduction in air pollutant emissions. In reference [11], the robust dynamic evolutionary optimization of the reactive power system in interconnected systems under fluctuating and uncertain wind power conditions was proposed. It has been suggested that leveraging the reactive power range embedded in wind farms can improve safety and optimality during the power system reactive power optimization process [12]. The reactive power optimization including interval uncertainty model was applied for developing a voltage control strategy to ensure that the state variables of a power grid reside within their safe operating limits [13]. The authors of [14] considered the hydrogen and PV as distributed generation and proposed a list of reactive power regulation strategies. However, the work presented above does not mention the participation of PV and EV in reactive power optimization. 

Some researchers have synthesized the active power generation and consumption as constraints and considered the cost of reactive power injection. The particle swarm optimization (PSO) was used to optimize the reactive power [2]. Similarly, aiming at the cost, the authors of [15] presented an in-depth study on the PV-Biomass hybrid independent power generation system in remote areas. Non-dominated sorting genetic algorithms III (NSGA-III) were proposed to address the reactive power optimization model which was established with the objective of minimizing system active power losses, controllable loads reduction, and PV active power reduction [16]. The authors of [17] discussed the reactive power based on capacitors allocation by using mathematical remora optimization algorithm. A data-driven model was employed to address the uncertain output of distributed generators for reactive power optimization in reference [18]. In reference [19], reactive power, the number of shunt capacitors and transformer taps were taken as optimization objectives and solved by the improved firefly algorithm. The authors of [20] pointed out that the reactive power control and actual power reduction of photovoltaic inverter could be effectively solved by a global sequential quadratic programming (SQP) approach method. Despite all this, these algorithms manifested the high consumption in time and instability in optimization.

By contrast, as a popular technology in recent years, deep learning (DL) may replace the traditional optimization process in the aspects of fitting data and improving the efficiency. The biggest feature of this method is that it can use a large amount of data for supervised learning and predict the optimization results in other cases via the previous optimization data set [21,22,23]. In this paper, five different multi-objective optimization algorithms are listed, and the same problem is optimized respectively. Through the training of DL, the optimization results of the five algorithms can fit the Pareto front (PF) like the previous optimization process. The fitted PF can be further optimized by correcting and supplementing some negative points.

Thus, the innovations of the proposed technique for reactive power optimization in this paper can be summarized as follows:

- In general, the reactive power optimization only considers the regulation of traditional equipment without the participation of PV systems or EVs, so that the reactive power regulation capacities of these new regulation sources are wasted. In this work, PV and EVs are simultaneously employed to participate in reactive power optimization in a distribution network, which can greatly decline the pressure of traditional reactive power regulation and improve the regulation flexibility and performance.
- To address the multi-objective reactive power optimization, the meta-heuristic based Pareto optimization algorithms easily result in a long computation time to acquire the high-quality Pareto optimal solutions. Besides, they easily lead to different Pareto front in different runs due to their random operators. In contrast, the proposed deep learning-based Pareto optimization algorithm can acquire the high-quality Pareto optimal solutions within a short computation time since it cannot experience multiple iterative operators. Moreover, it is a deterministic algorithm to guarantee a high optimization stability.

The rest of this paper is structured as follows. In Section 2, the model of PV and EVs connected to the distribution network will be explained. Section 3 will illustrate different algorithms simply which are applied on the model, and introduce the flow chart of the reactive power optimization. Section 4 presents the experimental results and analysis for different examples. Finally, the work will be concluded in Section 5.

## 2. Reactive Power Optimization of PV and EVs Connected to Distribution Network

### 2.1. Reactive Power Regulation Model of PV

**Figure 1:**
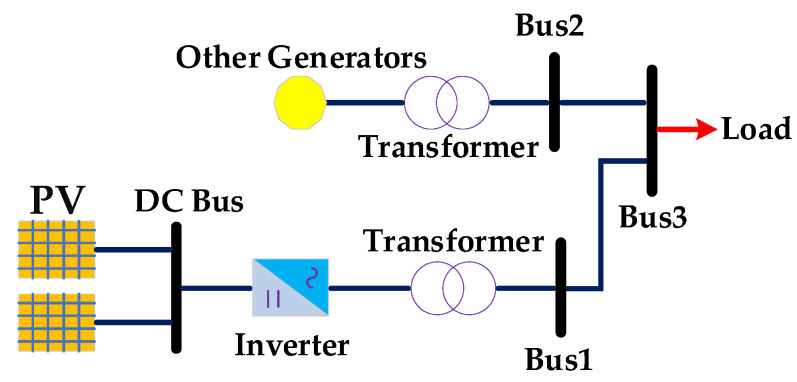
Schematic diagram of PV system reactive power regulation.

Generally, small photovoltaic equipment is usually connected to the distribution network. PV scattered at different nodes are characterized by high flexibility and can effectively improve the performance of power system [24,25]. When photons in solar radiation pass through the interior of the PV system, the equipment will release electrons to form the electric current. As shown in Figure 1, an inverter needs to be added since the direct current (DC) is released from the PV system when power is injected into the distribution network. Thus, the converted alternative current (AC) can be absorbed by the distribution network and redistributed to different loads [26]. PV systems are connected to PQ nodes or PV nodes of distribution network due to their ability to regulate reactive power. There are many factors affecting the power output of the PV system, which can be summarized as solar irradiation and real-time temperature [27]. The active power $P_{\mathrm{pv}}$ can be presented as follows:

(1) $$P_{\mathrm{pv}}=P_{\mathrm{pv}}^{\mathrm{base}}\left[1+\alpha_{\mathrm{pv}}\cdot \;\left(\;T-T_{\mathrm{ref}}\;\right)\;\right]\cdot \;\frac{s_{\mathrm{pv}}}{1000}$$

With the inverter is added behind the PV system, the reactive power regulation model is closely related to it. The output active power of the PV system will affect the range of reactive power directly in the current regulation model, as follows:

(2) $$\left\{\begin{array}{c} Q_{\mathrm{pv},\max}=\sqrt{\;{\left(\;S_{\mathrm{pv}}\;\right)}^{2}-{\left(\;P_{\mathrm{pv}}\;\right)}^{2}}\\ Q_{\mathrm{pv},\min}=-\sqrt{{\left(\;S_{\mathrm{pv}}\;\right)}^{2}-{\left(\;P_{\mathrm{pv}}\;\right)}^{2}} \end{array}\right.$$

### 2.2. Reactive Power Regulation Model of EVs

**Figure 2:**
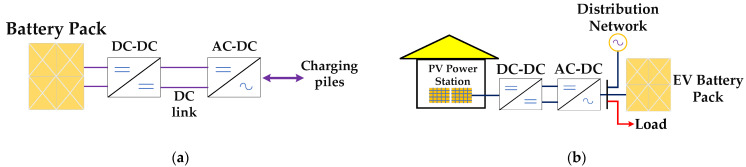
(**a**) Working schematic diagram of EV; (**b**) Schematic diagram of PV system impact mitigation by EVs.

EVs have basically been popularized in some big cities and their surrounding areas through some surveys. Therefore, the V2G technology of EVs can be used to assist the PV system. EVs can store more electric energy in the battery at the highest rate when the working efficiency of the PV system is too high. Similarly, EVs idled in the charging station can discharge to the distribution network at the highest rate during peak power consumption. The quantitative change will produce qualitative change and provide or absorb a large amount of power when there are a certain number of EVs connected to the distribution network [28]. The PV system and electric vehicle can affect the power grid as a pair of complementary modules to some extent. Figure 2 shows the principle of reactive power regulation complementary to the PV system when EVs are connected to distribution network [7]. In order to convert the AC transmitted from the distribution network into DC, a rectifier needs to be used for AC-DC conversion. The current converted into DC may be further filtered and the voltage may be changed through the DC-DC conversion. Finally, the optimized DC can be stored in the special battery of electric vehicle. Likewise, EVs can also transmit power to the distribution network through the opposite steps [29].

The input or output active power of EVs $P_{\mathrm{car}}$ is closely related to the converters they are connected to, as follows:

(3) $$P_{\mathrm{car}}=\frac{V_{s}V_{c}\sin\left(\delta \right)}{\omega L_{c}}$$

In this work, the active power of EVs is not the optimization variable in the multi-objective reactive power optimization in a distribution network. Therefore, the optimization model will be solved by assuming the fixed active power of EVs at each time period. In a practical application, the active power of EV can be acquired via the real-time data acquisition or power forecasting. During the transmission of EVs to the distribution network, the calculation of reactive power regulation range and principle is similar to that of the PV system. Its upper limits $Q_{\mathrm{car},\max}$ and lower limits $Q_{\mathrm{car},\min}$ of reactive power can be determined by the inverter and $P_{\mathrm{car}}$ as (4):

(4) $$\left\{\begin{array}{c} Q_{\mathrm{car},\max}=\sqrt{\;{\left(\;S_{\mathrm{car}}\;\right)}^{2}-{\left(\;P_{\mathrm{car}}\;\right)}^{2}}\\ Q_{\mathrm{car},\min}=-\sqrt{{\left(\;S_{\mathrm{car}}\;\right)}^{2}-{\left(\;P_{\mathrm{car}}\;\right)}^{2}} \end{array}\right.$$

### 2.3. Objective Function

The most important thing in the process of power generation and transmission is its economy and security, both of which are indispensable. Part of the energy will lose and convert into heat due to the resistance of the conductor when the electricity is transmitted through the transmission network. Besides, the surface density of field exceeds the breakdown strength of the surrounding air after the conductor is charged, resulting in corona loss of the thin layer of air around the conductor. Thus, line loss, which includes the loss of heat and corona loss, can be regarded as an important optimization goal related to the economy [30]. When DGs are connected to the distribution network, the reduction of transmission power on the distribution feeder and the reactive power output of PV system will cause high voltage at each load node on the distribution feeder and form voltage deviation. Voltage deviation is an important indicator of power quality. Excessive voltage deviation will do great harm to safety, stability, and economic operation [31]. The line loss minimization and voltage deviation minimization are regarded as a MOP. Finally, MOEAs are used to optimize the problem [32] and the neural networks are used to predict the optimization results. The formulas of the two objective functions are as follows (5):

(5) $$\left\{\begin{array}{c} \min\;f_{1}=\underset{i,j\in N_{L}}{\sum }\;g_{ij}\left(\;V_{i}^{2}+V_{j}^{2}-2V_{i}V_{j}\cos\theta_{ij}\;\right)\\ \min\;f_{2}=\underset{j\in N_{i}}{\sum }\;{\left(\;V_{j}-V_{j}^{*}\;\right)}^{2} \end{array}\right.$$

### 2.4. Constraint Condition

There will be some constraints when using MOEA to optimize the MOPs. They include the constraints of power flow in power system and the setting of some important parameter ranges in distribution network [33].

#### 2.4.1. Power Flow Equality Constraints

The conventional power flow equality constraints can be written as follows:

(6) $$\left\{\begin{array}{c} P_{Ga}-P_{Di}-V_{i}\underset{j\in N_{i}}{\sum }V_{j}\;\left(\;g_{ij}\cos\theta_{ij}\;+\;b_{ij}\sin\theta_{ij}\;\right)\;=0\;,\;i\in N_{0}\\ \;Q_{Ga}-Q_{Di}-V_{i}\underset{j\in N_{i}}{\sum }V_{j}\;\left(\;g_{ij}\sin\theta_{ij}-b_{ij}\cos\theta_{ij}\;\right)\;=0\;,\;i\in N_{\mathrm{PQ}} \end{array}\right.$$

#### 2.4.2. Generator Constraints

The generator outputs should satisfy the following constraints:

(7) $$\left\{\begin{array}{c} Q_{Ga}^{\min}\;\leq \;Q_{Ga}\;\leq \;Q_{Ga}^{\max}\;\;,\;a\in \;N_{G}\\ V_{Ga}^{\min}\;\leq \;V_{Ga}\;\leq \;V_{Ga}^{\max}\;\;,\;a\in \;N_{G} \end{array}\right.$$

#### 2.4.3. Reactive Power Compensation Device and Transformer Tap Constraints

The actions of reactive power compensation device and transformer tap should be limited within their bounds, as follows:

(8) $$\left\{\begin{array}{c} Q_{Cb}^{\min}\;\leq \;Q_{Cb}\;\leq \;Q_{Cb}^{\max}\;\;,\;\;b\in \;N_{c}\\ T_{h}^{\min}\;\leq \;T_{h}\;\leq \;T_{h}^{\max}\;\;,\;\;h\in \;N_{T} \end{array}\right.$$

#### 2.4.4. Security Constraints

To guarantee a safe operation, the node voltage and line transmission power should satisfy the following constraints:

(9) $$\left\{\begin{array}{c} V_{i}^{\min}\;\leq \;V_{i}\;\leq \;V_{i}^{\max}\;\;,\;\;i\in \;N_{\mathrm{PQ}}\\ \left|\;S_{l}\;\right|\;\leq \;S_{l}^{\max}\;\;,\;\;l\in \;N_{L} \end{array}\right.$$

Note that the presented multi-objective reactive power optimization in Equations (5)–(9) is a NP-hard optimization with a high non-linearity and multiple local optimums [34]. In general, it is very difficult to acquire the global solutions for this problem. Hence, the proposed method is used to acquire the high-quality local optimums with a high probability.

## 3. Optimized Variable Prediction and the Process of Reactive Power Regulation Model

### 3.1. Overview of PREA, SPEA2, NSGA-II, NSGA-III and TOP

The promising-region-based evolutionary many-objective algorithm (PREA) [35] investigates the properties of ratio and difference-based indicators under the Minkovsky distance. PREA with the ratio-based indicator is proposed by the researchers according to the ratio-based indicator with infinite norm. This new MOEA proposes an individual selection strategy based on parallel distance to ensure the diversity of the population in the algorithm. PREA has relatively high dominance in the optimization problems with 3–20 objectives from the experimental results. Having stronger robustness is the characteristics of PREA compared with the current advanced MOEA.

The strength Pareto evolutionary algorithm 2 (SPEA2) [36] stores the nondominated solutions in another continuously updated population. Then it computes the fitness according to the number of nondominated solutions that an individual independently dominates. A cluster analysis process is added for the sake of reducing the nondominated solution set without destroying its characteristics. In addition, Pareto dominance is used to preserve population diversity in SPEA2. In some MOPs, SPEA2 shows excellent optimization ability. Path planning simulation can be addressed by the traditional hybrid target method and the improved SPEA2 based on a local search.

A fast non-dominated sorting genetic algorithm II (NSGA-II) is proposed in [37] to reduce the complexity of calculating nondominated orders. An elite strategy is introduced and the sampling space is expanded in NSGA-II. Such approaches are conducive to maintaining the excellent individuals in the parent generation, ensuring that those great individuals will not be discarded in the process of evolution. Therefore, the accuracy of optimization results can be highly improved. The best individuals will not be lost, and the population level can be rapidly improved by storing all individuals in layers. NSGA-II takes the crowding degree as the comparison criterion between individuals in the population. It has the characteristic that the population individuals in the quasi Pareto domain can be evenly extended to the whole Pareto domain, which ensures the diversity of the population.

NSGA-III [38] can be regarded as an improved algorithm of NSGA-II. With the development of technology, optimization problems become more and more complex. In order to adapt to the development of MOPs, NSGA-III is proposed and widely used to deal with high-dimensional problems with objective dimension greater than 3. The nondominated individuals of the population will increase exponentially in high-dimensional problems. Thus, an issue that it is difficult to distinguish individuals by Pareto dominance will be exposed. The decomposition-based algorithm is proposed in NSGA-III to set reference points and then address this kind of problem.

Two-phase framework (ToP) [39] is the most recently published algorithm that mainly handles constrained multiobjective optimization problems (CMOPs). Without considering the decision constraints and the objective constraints, CMOPs may become easier but not practical. In order to improve the performance of current algorithms in dealing with such problems, ToP is proposed to obtain feasible solutions with more uniform distribution and better convergence.

### 3.2. Application of DL in Reactive Power Optimization of Distribution Network

In this paper, deep deconvolutional neural network (DDNN) is applied to acquire the knowledge between the reactive power regulation command and the Pareto optimal solutions based on the standardized data. DDNN, which is based on a deep neural network (DNN) and developed from a fully connected neural network, improves the fault tolerance rate and data analysis ability of data processing. For good measure, DDNN will avoid an excessive co-adaptation between different neurons to a large degree. Then, the new network that is more robust than before can be applied to handle the overfitting problem. Thus, according to the reactive power regulation command, the trained DDNN can directly generate the approximate PF, as follows (10):

(10) $${\hat{x}}_{\mathrm{nt}}^{*}=g_{\mathrm{DDNN}}\left(W,b,\Delta Q\right)$$

To obtain multiple sets of data for prediction with DL, a variety of intelligent algorithms are applied to the optimization of the problem. Both continuous variables and discrete variables will exist in the process of reactive power optimization. Continuous variables can be iterated according to normal optimization, and discrete variables need to be rounded by continuous spatial values. Besides, the fitness function of each algorithm should also add a penalty mechanism to ensure that the optimization results meet the above constraints, as follows (11):

(11) $$f_{\mathrm{fit},d}\left(\;x^{i}\;\right)=f_{d}\left(\;x^{i}\;\right)+\eta q,\;d\;\in \;D\;$$

The advantages of different algorithms will be reflected in the PF. Thus, the prediction can concentrate and use the optimized data obtained by the algorithms with different characteristics. The method that calculates the Euclidean distance between the infeasible solution and the feasible solution is common in correcting the infeasible solution. Consequently, the points on the PF can be sorted and pruned before the data training. In order to better extract the characteristics of the optimized data obtained by multiple algorithms, the number of layers of the DL network will be set to five in this work. Each layer is set as a fully connected layer, and the dimension of the input features are different. 

**Figure 3:**
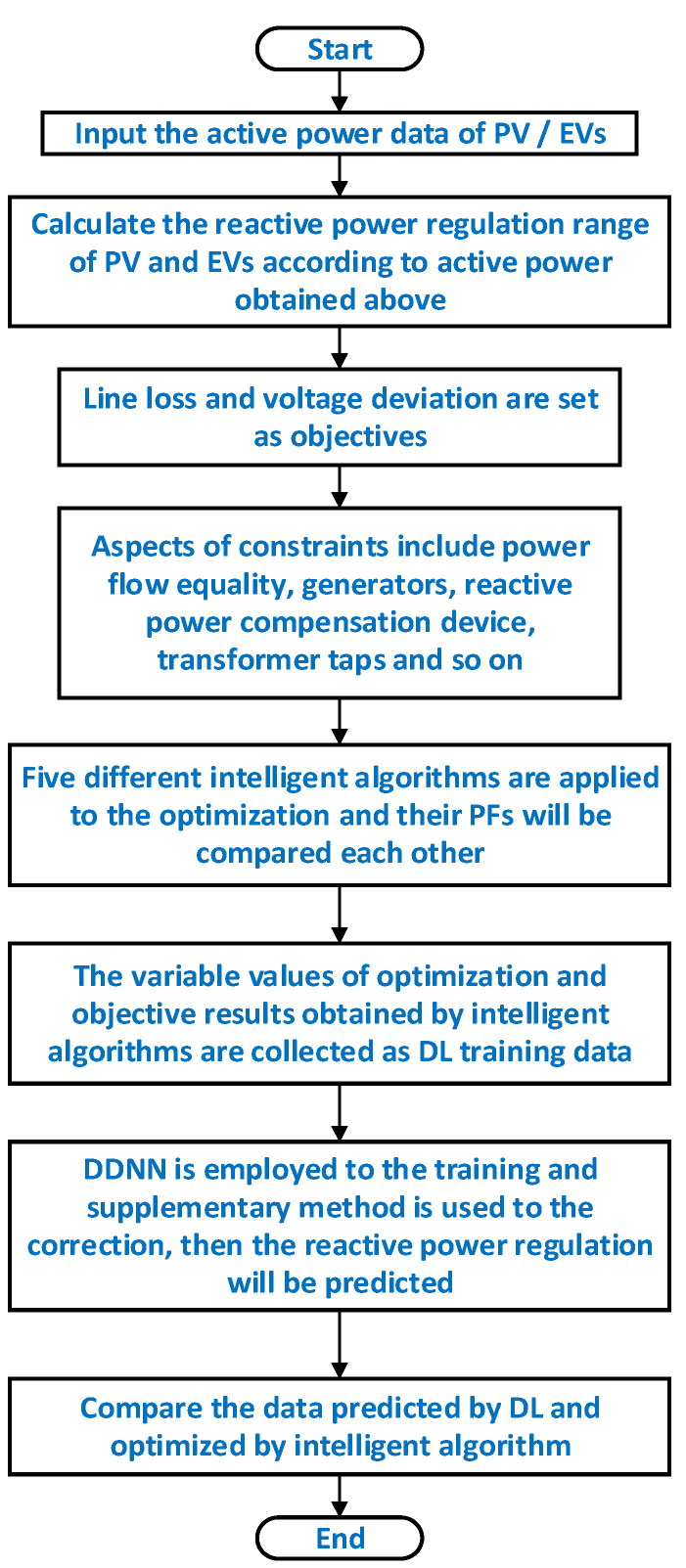
Flow chart of prediction with DL.

The process of multi-objective reactive power optimization can be summarized as Figure 3. Firstly, the basic operating data of the distribution network including the active power data are taken as the inputs. Then, the reactive power regulation range of PV and EVs can be evaluated according to their current active power. Meanwhile, the objective functions and constraints will be determined based on the optimization model constructed above. Five different intelligent algorithms are used to generate the training data for DDNN. Hence, DDNN can be trained by the data from the optimization results, and then it can be adopted to rapidly acquire the Pareto optimal solutions for different tasks of multi-objective reactive power optimization.

## 4. Example Analysis

### 4.1. Simulation Model

**Figure 4:**
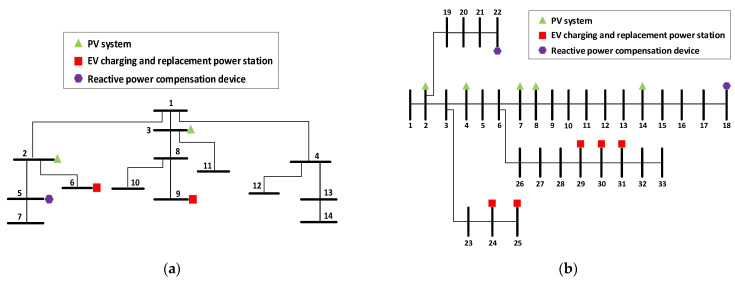
(**a**) Topologies of IEEE 14-bus system; (**b**) Topologies of IEEE 33-bus system.

In this work, IEEE 14-bus and IEEE 33-bus distribution network will be used to test the different algorithms. The topology of the two models are shown in Figure 4. In the IEEE 14-bus system, the installed capacity of PV is set as 1 MW, while the capacity of EVs charging and replacement power station can be set as 100 kW. Only two PV in small scale and two EV charging and exchange stations were simulated in the experiment due to the limited number of nodes. The location of connected nodes can be obtained from Figure 4. Hence, two groups of reactive power regulation ranges need to be optimized can be obtained from active power of PV and the EVs power station. Besides, 1 reactive power compensation device and 3 taps of transformer are set, each of which will have 5 gears {0.98, 1.00, 1.02, 1.04, 1.06} pu for adjustment respectively. Similarly, the capacity of PV and EV station are set as 1 MW and 100 kW, respectively. PV or EV stations connected to the distribution network will increase accordingly while the number of nodes increases. Therefore, 5 different reactive power regulation ranges can be obtained by the 5 PV and 5 EV stations respectively in the simulation. At the same time, 2 reactive power compensation devices and 5 taps of transformer are set.

Generally, the main influencing factors of PV output power are current temperature and solar irradiation. Thus, different solar irradiation will be set in different PV to observe its impact on the reactive power regulation range. In addition, each EV charging and replacement station accommodates 10 EVs, and the residual power of each EV is 0–10 kW by considering actual optimal scheduling problem of EVs [40]. The range of total power can be simply added to 0–100 kW. Table 1 and Table 2 show the parameters range of PV and EV station in IEEE 14-bus and IEEE 33-bus, respectively.

It is necessary to ensure that the initialization conditions are the same when the different intelligent algorithms for optimization are used. The population number and maximum iteration of the optimization process are set to 50 in order to simplify the calculation and accelerate the experimental progress. In the process of optimization, five different algorithms will operate in the same environment. 

### 4.2. Analysis of Experiment Results

**Figure 5:**
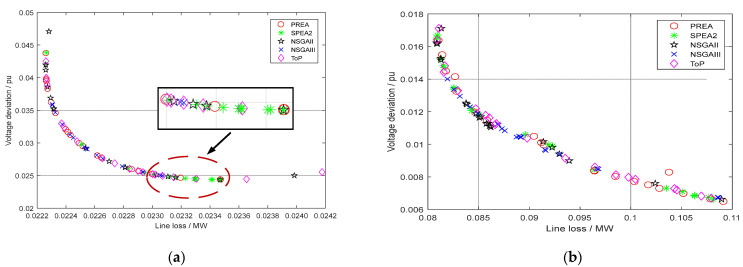
(**a**) PF comparison of different algorithms in IEEE 14-bus system; (**b**) PF comparison of different algorithms in IEEE 33-bus system.

Figure 5 shows the acquired PFs of IEEE 14-bus and IEEE 33-bus systems in the case of this model, respectively. The abscissa of PF is set as the line loss of distribution network, and the ordinate is set as the voltage deviation. NSGA-II and ToP have a wider PF than other algorithms on the IEEE 14-bus system. This reveals that these two algorithms can implement a wider exploration for multi-objective reactive power optimization on the IEEE 14-bus system. The optimization results of NSGA-III will be densely distributed in the middle of the front, which results from the strong exploitation for a local area. However, the performance of SPEA2 is somewhat unsatisfactory. Most of the optimization results prefer to minimize the voltage deviation in the locally enlarged figure. In contrast, the distribution range of line loss is much smaller. The PF distinction on the IEEE 33-bus system can be more obvious than that on the IEEE 14-bus system from Figure 5b. PREA performs relatively poorly among all the algorithms. It can be found that slight dominated solutions are distributed near the two ends of the PF in many simulations. The optimization results of SPEA2 in this system still have the problem of uneven distribution due to the blind selection from the Pareto repository. Many solutions only perform well in a single objective minimization, but it is difficult to achieve the balance of multi-objective minimization. NSGA-II and NSGA-III perform better in the optimization value. Nonetheless, these two algorithms still have the issue of small distribution range compared with other algorithms.

The solution differences between DL and other five meta-heuristic-based Pareto optimization algorithms are presented in Table 3 and Table 4. It can be found that the range of PF obtained by DL is slightly narrower than that by other algorithms. It demonstrates that, with DL, it is difficult to achieve a high generalization for all the Pareto optimal solutions. Even though there is a gap of maximum and minimum value among DL and other algorithms, the average line loss and voltage obtained by DL are almost the smallest among all the algorithms since its training data is generated from multiple algorithms. Besides, when it comes to the economic benefits of optimization, this model of reactive power regulation embodies its advantages. In the IEEE 14-bus system, the electric energy saved from line loss is about 192 kWh per day, i.e., the cost saved in one day is $19.2 when the electricity price is set to $0.1 per kWh. Furthermore, the expenses reduced from optimization can be calculated as $16.7 per day in the IEEE 33-bus system.

In addition, the five algorithms and DL have almost the same goal of minimizing line loss in IEEE 14-bus system from Table 3. However, there is little difference in the results of voltage deviation minimization and optimization among various algorithms in the IEEE 33-bus system from Table 4. Besides, the conclusions observed in the graph can also be obtained from the table data. The PF of NSGA-II shows excellent scalability compared with other algorithms in the 14-bus system while its multi-objective minimization ability is incisively and vividly demonstrated in the 33-bus system. The simulation of ToP can get relatively stable values under any system model from the average data. It must have great advantages in some practical applications that highlight stability compared with other algorithms with large numerical changes in optimization results. In general, the prediction results of DL have little difference from the five algorithms. Both the width of PF and optimization degree can basically reach the optimal value of various intelligent algorithms.

Take the IEEE 33-bus system as an example to verify the prediction effect of DL in this kind of model. Two PV and three EV charging and replacement power stations are set as same as the above experiments. Every PV can output 10 kinds of active power values, and the output data of EV charging and replacement stations are set randomly. The above five algorithms are used to optimize the model respectively, and a large amount of optimized reactive power data is obtained through the above active power. The optimized results in each case will be used as samples for DL training. Offline training only takes a few minutes to get the same number of solutions as the number of data groups. Then the training results integrated comprehensively by the five algorithms will be used in the same case. Some dominated solutions will be deleted, and some virtual solutions will be supplemented, so that the PF will become more smooth than before. There is no doubt that the supplementary solution must remain within the constraints. Thus, a set of solutions with five different excellent algorithms can be used for prediction.

**Figure 6:**
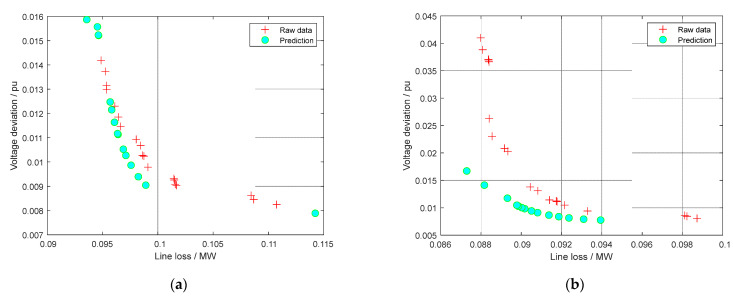
Examples of DL prediction and raw data in IEEE-33 bus system. (**a**) Example 1; (**b**) Example 2.

The comparison between the prediction results of DL and the integrated results from five algorithms is shown in Figure 6. It can be seen that the predicted results of DL can basically reach or even exceed the optimization results integrated by five algorithms. Firstly, DL can extend the PF range compared with the integrated PF by five algorithms in Figure 6a, which resulted from the effective generalization formed in network training. Secondly, DL can further improve the PF quality compared to the original integrated PF, as shown in Figure 6b. This verifies that the training data from different optimization tasks can enhance the Pareto optimization performance of DL due to the high similarity between them. Furthermore, the computation time consumed by different methods is provided in Table 5. The computation time of all the algorithms except DL is close because their computation time is mainly determined by the population size and maximum iteration number, where these two parameters are set to be the same values for each algorithm. Note that the computation time of DL is the shortest among all the algorithms as it can directly generate the Pareto optimal solutions without multiple iterative operators. In particular, the computation time of NSGA-II is about 79.58 times that of DL.

## 5. Conclusions

In this work, a novel DL based Pareto optimization method is proposed for multi-objective reactive power optimization in a distribution network with PV and EVs, which contains the following contributions:

(1) By taking the participation of PV and EVs into account, the reactive regulation burden of the distribution network can be effectively reduced. As a result, the operation economy and the voltage quality of the distribution network can be further improved. Simulation results demonstrate that the line loss can be reduced by 25.2% by the proposed method compared to that without the participation of PV and EVs on the IEEE 14-bus system, and 7.7% on the IEEE 33-bus system. In addition, the voltage deviation can be reduced by 0.16% and 0.38% on the IEEE 14-bus and IEEE 33-bus systems, respectively.

(2) The proposed technique for multi-objective reactive power optimization is verified on IEEE 14-bus and IEEE 33-bus systems, which is compared with five different intelligent algorithms. Simulation results show that the proposed DL method can rapidly acquire a high-quality PF, while another five algorithms can complete the optimization task well, and the Pareto optimal solutions have little difference in the degree of optimization. Particularly, the computation time of DL is only 1.26% of that by NSGA-II on the IEEE 33-bus system, while the average line loss and voltage deviation of all the Pareto optimal solutions obtained by DL are the smallest among all the algorithms.

Although the proposed DL based multi-objective reactive power optimization can perform well on the test systems, it still easily faces two main challenges. Firstly, it should spend a long computation time on the data acquisition and network training. Secondly, it will easily lead to multiple infeasible solutions due to the poor generalization of DL with insufficient data. To handle these two problems, our future work will focus on the historical data utilization and the generalization for DL.

## Figures and Tables

**Table 1:** Parameters of PV and EV stations in the IEEE 14-bus distribution system.

Number		$\mathrm{Solar}\;\mathrm{Irradiation}\;(W\cdot m^{-2})$	Active Power (kW)	Reactive Power Range (kVar)
PV	1	600	12	[−17.23, 17.23]
	2	700	14	[−15.65, 15.65]
EV	1		−56.76	[−82.33, 82.33]
	2		−49.16	[−87.08, 87.08]

**Table 2:** Parameters of PV and EV stations in the IEEE 33-bus distribution system.

Number		$\mathrm{Solar}\;\mathrm{Irradiation}\;(W\cdot m^{-2})$	Active Power (kW)	Reactive Power Range (kVar)
PV	1	600	12	[−17.23, 17.23]
	2	700	14	[−15.65, 15.65]
	3	800	16	[−13.60, 13.60]
	4	900	18	[−10.82, 10.82]
	5	1000	20	[−6.40, 6.40]
EV	1		−48.38	[−87.52, 87.52]
	2		−53.31	[−84.61, 84.61]
	3		−52.14	[−85.33, 85.33]
	4		64.99	[−76.00, 76.00]
	5		−44.67	[−89.47, 89.47]

**Table 3:** Statistical results of PFs obtained in the IEEE 14-bus system.

Objective	Algorithm	Minimum	Maximum	Average
Line loss/MW	PREA	0.0223	0.0235	0.0226
	SPEA2	0.0223	0.0235	0.0231
	NSGA-II	0.0223	0.0240	0.0227
	NSGA-III	0.0223	0.0231	0.0226
	ToP	0.0223	0.0242	0.0228
	DL	0.0236	0.0251	0.0240
Voltage deviation/pu	PREA	0.0248	0.0438	0.0316
	SPEA2	0.0244	0.0438	0.0268
	NSGA-II	0.0244	0.0471	0.0327
	NSGA-III	0.0250	0.0359	0.0292
	ToP	0.0245	0.0425	0.0298
	DL	0.0245	0.0421	0.0308

**Table 4:** Statistical results of PFs obtained in the IEEE 33-bus system.

Objective	Algorithm	Minimum	Maximum	Average
Line loss/MW	PREA	0.0810	0.1091	0.0938
	SPEA2	0.0809	0.1081	0.0916
	NSGA-II	0.0813	0.1089	0.0877
	NSGA-III	0.0819	0.1086	0.0904
	ToP	0.0809	0.1079	0.0920
	DL	0.0873	0.0939	0.0906
Voltage deviation/pu	PREA	0.0065	0.0164	0.0102
	SPEA2	0.0066	0.0167	0.0111
	NSGA-II	0.0066	0.0171	0.0119
	NSGA-III	0.0067	0.0140	0.0105
	ToP	0.0067	0.0171	0.0107
	DL	0.0077	0.0167	0.0102

**Table 5:** Comparison of time required for different algorithm optimization and DL prediction in IEEE-33 bus system.

Algorithm	Average Time/Seconds
PREA	7.77
SPEA2	8.67
NSGA-II	9.55
NSGA-III	9.03
ToP	9.70
DL	0.12

## Data Availability

Not applicable.

## Nomenclature

Variable	
$P_{\mathrm{pv}}$	active power of PV system
$P_{\mathrm{car}}$	input or output active power of EVs
*T*	real-time temperature
$s_{\mathrm{pv}}$	solar irradiation at the current time
$Q_{\mathrm{pv},\max}$	upper limits of adjustable reactive power range of PV system
$Q_{\mathrm{pv},\min}$	lower limits of adjustable reactive power range of PV system
$Q_{\mathrm{car},\max}$	upper limits of adjustable reactive power range of EVs
$Q_{\mathrm{car},\min}$	lower limits of adjustable reactive power range of EVs
δ	phase difference between $V_{s}$ and $V_{c}$
ω	angular frequency of the sine wave in the AC system
$f_{1}$	line loss
$f_{2}$	voltage deviation
$V_{i}$	voltage amplitude of the *i*th node
$V_{j}$	voltage amplitude of the *j*th node
$\theta_{ij}$	phase angle difference between the *i*th and *j*th nodes
$P_{Ga}$	active power of the *a*th generation node
$Q_{Ga}$	reactive power of the *a*th generation node
$P_{Di}$	active power demand of the *i*th node
$Q_{Di}$	reactive power demand of the *i*th node
$Q_{Ga}^{\min}$	lower limits of reactive power regulation of the *a*th generator
$Q_{Ga}^{\max}$	upper limits of reactive power regulation of the *a*th generator
$Q_{Ga}$	reactive power currently input into the grid by the *a*th generator
$V_{Ga}^{\min}$	lower limits of output voltage of the *a*th generator
$V_{Ga}^{\max}$	upper limits of output voltage of the *a*th generator
$V_{Ga}$	current output voltage of the *a*th generator
$Q_{Cb}^{\min}$	lower limit of the capacity of the *b*th reactive power compensation device
$Q_{Cb}^{\max}$	upper limit of the capacity of the *b*th reactive power compensation device
$T_{h}^{\min}$	lower limit of the regulation range of the *h*th transformer tap
$T_{h}^{\max}$	upper limit of the regulation range of the *h*th transformer tap
$V_{i}^{\min}$	lower voltage limits of the *i*th node
$V_{i}^{\max}$	upper voltage limits of the *i*th node
$S_{l}$	apparent power of the *l*th line
${\hat{x}}_{nt}^{*}$	approximate Pareto optimal solutions for the new task
ΔQ	reactive power regulation command of a new task
$f_{d}\left(\;x^{i}\;\right)$	objective function value
$f_{fit,d}\left(\;x^{i}\;\right)$	value of fitness function
Parameters	
$P_{\mathrm{pv}}^{\mathrm{base}}$	total rated power of PV system
$\alpha_{\mathrm{pv}}$	temperature conversion coefficient
$T_{\mathrm{ref}}$	reference temperature
$V_{s}$	grid voltage
$V_{c}$	charging piles voltage
$S_{\mathrm{pv}}$	capacity of the inverter
$S_{\mathrm{car}}$	capacity of charging piles inverter
$L_{c}$	simplified inductance
$g_{ij}$	admittance between the *i*th and *j*th nodes
$N_{i}$	total node set
$N_{L}$	all branch set
$N_{0}$	node set except the balance node
$N_{\mathrm{PQ}}$	PQ node set
$V_{j}^{*}$	rated voltage of the *j*th node
$N_{G}$	generator set
$N_{c}$	set of reactive power compensation devices
$N_{T}$	set of transformer taps
$b_{ij}$	susceptance between the *i*th and *j*th nodes
$S_{l}^{\max}$	transmission power limit of the *l*th line
$g_{\mathrm{DDNN}}$	trained DDNN network
*W*	weight set
*B*	bias set
*D*	set of objective functions
η	penalty coefficient
Indices	
*i*	index of node
*j*	index of node
*a*	index of generator
*b*	index of reactive power compensation device
*h*	index of transformer tap
*l*	index of bus
*d*	index of the objective functions value
*q*	index of the objective
Abbreviations	
RES	renewable energy sources
PV	photovoltaic
DG	distributed generations
DER	distributed energy resources
V2G	vehicle-to-grid
MOP	multi-objective problem
MOEA	multi-objective evolution algorithm
PF	Pareto front
SQP	sequential quadratic programming
DL	deep learning
DC	direct current
AC	alternative current
PSO	particle swarm optimization
NSGA-II	non-dominated sorting genetic algorithms II
NSGA-III	non-dominated sorting genetic algorithms III
PREA	promising-region-based evolutionary many-objective algorithm
SPEA2	strength Pareto evolutionary algorithm 2
ToP	two-phase framework
DDNN	deep deconvolutional neural network
DNN	deep neural network
